# Computed Tomography-Guided Percutaneous Microwave Ablation for Renal Cell Carcinoma: Impact of Tumor Size on the Progression Survival Rates

**DOI:** 10.3390/diagnostics11091618

**Published:** 2021-09-04

**Authors:** Evgenia Efthymiou, Argyris Siatelis, Christos Liakouras, Georgios Makris, Michael Chrisofos, Alexis Kelekis, Elias Brountzos, Nikolaos Kelekis, Dimitrios Filippiadis

**Affiliations:** 1 2nd Department of Radiology, Medical School, University General Hospital “ATTIKON”, National and Kapodistrian University of Athens, 12462 Athens, Greece; efthymiouevgenia@gmail.com (E.E.); akelekis@med.uoa.gr (A.K.); ebrountz@med.uoa.gr (E.B.); kelnik@med.uoa.gr (N.K.); 2 C Urology Clinic, Medical School, University General Hospital “ATTIKON”, National and Kapodistrian University of Athens, 12462 Athens, Greece; argysiat@yahoo.gr (A.S.); drliakouras@hotmail.com (C.L.); drmakrisgiorgio@gmail.com (G.M.); mxchris@yahoo.com (M.C.)

**Keywords:** microwave ablation, computed tomography, renal cell carcinoma

## Abstract

The aim of the present study was to evaluate the safety and efficacy of computed tomography (CT)-guided percutaneous microwave ablation (MWA) of renal cell carcinoma (RCC) along with identifying prognostic factors affecting the progression survival rate. Institutional database retrospective research identified 69 patients with a biopsy proven solitary T1a (82.6%) or TIb (17.4%) RCC who have underwent percutaneous CT-guided MWA. Kaplan–Meier survival estimates for events were graphed and Cox regression analysis was conducted. Mean patient age was 70.4 ± 11.5 years. Mean size of the lesions was 3 ± 1.3 cm. Mean follow up time was 35.6 months (SD = 21.1). The mean progression free survival time from last ablation was 84.2 months. For T1a tumors, the cumulative progression free survival rate for 1, 6, 12 and 36 months were 100% (SE = 0%), 91.2% (SE = 3.7%), 91.2% (SE = 3.7%) and 87.5% (SE = 4.4%); the recurrence free survival rate for T1a RCC was 94.9%. For T1b tumors, the cumulative progression free survival rate for 1, 6, 12 and 36 months were 100% (SE = 0%), 63.6% (SE = 14.5%), 63.6% (SE = 14.5%) and 63.6% (SE = 14.5%). Grade 1 complications were recorded in 5 (7.2%) patients. Significantly greater hazard for progression was found in cases with a tumor size > 4 cm (HR = 9.09, *p* = 0.048). No statistically important difference regarding tumor progression was recorded between T1a tumors with a diameter ≤3 cm and >3 cm. In summary, the results of the present study show that CT guided percutaneous MWA is an effective technique for treatment of T1a renal cell carcinomas, irrespective of tumor size. T1b tumors were associated with higher progression rates.

## 1. Introduction

Renal resection on terms of either radical or partial nephrectomy is a well-established local cure for the management of stage T1a and T1b RCC [1,2,3,4,5,6,7,8]. However, the advancement and the effective application of percutaneous ablation techniques in overall cancer care render them an efficient alternative and an attractive solution in the treatment of RCC. Percutaneous ablation is a nephron-sparing technique with similar metastasis-free survival and cancer-specific survival rates when compared to partial nephrectomy, with additional benefits of a shorter hospitalization and recovery time [9]. Alam et al. has reported that throughout a 7 years follow-up period, percutaneous ablation, radical or partial nephrectomy resulted in almost a 100% cancer-specific survival rate without any difference between the three treatment arms [10]. International guidelines advocate application of percutaneous ablation for RCC as an alternative therapeutic option for the management of localized masses with a diameter ≤3 cm, when complete ablation is technically feasible, for selected and counseled patients [4,5,6,7,8,9,11]. Although there is an extensive and thorough literature regarding the application of radiofrequency ablation (RFA) and cryoablation (CA) in renal tumors, the respective literature for MWA remains limited. When compared to RFA, MWA is less affected by the heat-sink effect, rendering the technique (at least in theory) ideal for the hypervascular renal tumors [12,13,14]. In theory, the high perfusion (4 times that of the liver) and potential heat dissipation of the kidney may alter the bio-heat equation, requiring a less sensitive to heat-sink effect treatment method for ablation.

The purpose of the present study is to evaluate the safety and efficacy of computed tomography-guided percutaneous microwave ablation (MWA) of renal cell carcinoma (RCC), along with identifying prognostic factors affecting the progression survival rate.

## 2. Materials and Methods

### 2.1. Patient Selection and Evaluation

Institutional Review Board approval was obtained for this single centered database research (2021032853/18 September 2020) with a waiver of informed consent. Institutional database research from 2013 till 2020 identified 69 RCC patients who were treated by CT-guided MWA. All included lesions should have been evaluable for a minimum of 6 months follow-up. Indications for MWA included RCC patients (T1a or T1b stage) confirmed by prior biopsy, a Karnofsky Performance Scale (KPS) score >60, coagulation parameters within normal limits and a life expectancy of >3 months. Contraindications for ablation included uncontrollable primary or metastatic disease outside the kidney, non-compliance of patients, uncontrollable INR, systemic or local infection, expected survival <3 months, an ECOG score >3 and the presence of a medical or psychiatric illness that would preclude informed consent of follow-up. All patients were evaluated in a multidisciplinary tumor board and referred for thermal ablation by consensus decision of urologists, medical oncologists and interventional radiologists. The patients were fully informed about the procedure, the possible complications and the surgical and medical alternatives available; informed written consent for percutaneous ablation was obtained in all cases. Each patient underwent laboratory tests (including renal function and coagulation tests) at least 24 h prior to the percutaneous ablation session. All MWA were performed by the same interventional radiologist, with over 10 years of experience with image-guided thermal ablation of RCC.

### 2.2. Percutaneous Computed Tomography-Guided Microwave Ablation

According to Infection Division of Pathology Department in each patient was injected intravenously with a dose of antibiotics [Cefuroxime 1.5 g, GlaxoSmithKline ABEE, Athens, Greece)] 45–60 min before the microwave ablation session. Blood thinning medications were held according to CIRSE guidelines [15]. Percutaneous microwave ablation was always performed in an inpatient setting under local anesthesia [10 cc of 2% Lidocaine Hydrochloric (Xylozan, DEMO ABEE, Athens, Greece)] on skin and subcutaneous tissues) and intravenous analgesia [30 min prior to ablation, 2 mL of tramadol 100 mg (Tramal 100 mg Vianex AE, Athens, Greece) were injected intravenously and diluted in 100 mL normal saline, whilst 100 mL of paracetamol 1 g (Fresenius Kabi Hellas AE, Athens, Greece)were administered during the ablation session to treat intra-procedural pain]. Ancillary procedures included ureteral stent placement, which was applied in select cases at the operator’s discretion aiming to reduce the potential risk of thermal non-target injury during MWA. A ureteral stent was placed by urologists on the day of the procedure. Computed Tomography guidance with sequential scanning (120 Kv, 240 mAs and 2 mm slice thickness) was used for planning, targeting and intra-procedural modification during MWA session. Under extended local sterility, microwave ablation was performed with a percutaneous approach in all cases. After the initial CT scan, skin entry point was selected. Depending on the size of the lesion, 1 or more microwave antennae (16 Gauge, 2.45 GHz AMICA^TM^ system, HS HOSPITAL SERVICE SpA, Rome, Italy) was/were inserted in the lesion of interest and the approach was evaluated with sequential CT scans. Once in the correct location, ablation session was performed according to data provided in charts for renal tumor microwave ablation obtained in human studies concerning the energy amount (watt), duration (minutes) and resultant ablation volume (centimeters) [16]. The goal was to ablate the whole lesion, including a circumferential zone of normal renal parenchyma and/or extra-renal fat, for at least 0.5 cm. Axial, coronal and sagittal images were reformatted intermittently to further evaluate the safety and efficacy of the session. Contrast enhanced computed tomography assessed any potential immediate complications at the end of the microwave ablation treatment. In the case of a lack of any advert effects, patients were discharged the following day.

### 2.3. Outcome Measures

Technical success was defined as successful completion of the planned microwave ablation of RCC. Treatment outcome was reported based on standard reporting criteria [17]. Patients had a follow up imaging with either contrast enhanced CT or MRI at 1, 3, 6 and 12 months after the ablation and every year after, usually combined with a consultation. Patients with a recurrent or residual disease were consulted for their options.

Patient demographics (age, sex) as well as tumor characteristics, microwave technique, pattern of recurrence and survival rate were evaluated. Prior to this, intra-procedural and follow up imaging were also evaluated. Primary technical success was defined as complete tumor necrosis after a single microwave ablation session with no evidence of tumor remnant or recurrence on subsequent cross-sectional imaging; secondary technical success rates were defined as complete tumor necrosis after a repeat microwave ablation of residual or recurrent disease [18]. Progression-free survival was defined as the time interval post MWA without evidence of local recurrence. The recurrence free survival rate was defined as the time elapsed between the intervention and any recurrence (local, regional or distant). Definition of complications was assigned according to the Cardiovascular and Interventional Radiological Society of Europe (CIRSE) classification system [19]. Tumor size category was referred to using the AUA guideline [6] and TNM (T1aN0M0 or T1bN0M0) staging system (<3 cm, 3.1–4 cm or ≥4 cm).

### 2.4. Statistical Analysis

Quantitative variables were expressed as mean values (SD and median (interquartile range), while qualitative variables were expressed as absolute and relative frequencies. Life table analyses were used to calculate a cumulative progression free survival rate (standard errors) for specific time intervals. Kaplan–Meier survival estimates for progression events were graphed over the follow-up period. The prognostic value of each variable was first assessed by univariate Cox regression analysis. Then all independent variables were included in the multivariate Cox proportional-hazard model in order to determine the predictors for disease progression. The assumption of proportional hazards was evaluated by testing for interaction with a continuous time variable. All reported *p* values are two-tailed. Statistical significance was set at *p* < 0.05 and analyses were conducted using SPSS statistical software (version 22.0).

## 3. Results

Patient and lesion demographics are reported in Table 1 and Table 2. Tumor stage was T1a (82.6%) or T1b (17.4%), (Table 1). The mean size of the lesions was 3 ± 1.3 cm, with maximum tumor size ranged from 1 to 6 cm. The mean follow up time was 35.6 months (SD = 21.1).

All procedures were performed under local anesthesia and conscious sedation and were well tolerated by all the patients. On a per lesion basis, as far as T1a RCCs were concerned, tumor remnants were noticed at one month follow up in three patients (3/57) (primary technical success 94.7%); all three patients were re-treated with an ablation session and no tumor remnant was depicted in the subsequent imaging follow-up (secondary technical success 100%). As far as T1b RCCs were concerned, tumor remnants were noticed at the one month follow up in four patients (4/12) patients (primary technical success 66.7%): 2/4 patients were re-treated with ablation combined with trans-arterial embolization, one patient was re-treated with ablation only and one patient refused any further treatment. Tumor recurrence was noted in 2/69 T1a clear cell RCC patients (2.9%) at one year of follow-up; both patients underwent a second ablation. A metastatic lesion was depicted in 2/69 (2.9%) patients, with T1a (clear cell RCC-metastatic lesion to the sacral bone was depicted 12 months post therapy and treated with ablation combined with sacroplasty) and T1b tumors (lesion was encountered in iliac bone 5 months post therapy and treated with ablation), respectively. Grade I self-limited complications included limited perinephric haematomas (n = 4) and small urinoma (n = 1) requiring nothing but observation; these complications were recorded in 5/69 (7.2%) patients (4/5 with T1a and 1/5 with T1b RCCs). Although evaluation of renal function was not included in the objectives of the study, all laboratory work-up (including urea and creatinine values) prior to contrast enhanced axial imaging during the follow-up period showed a lack of significant changes and deterioration.

**Figure 1:**
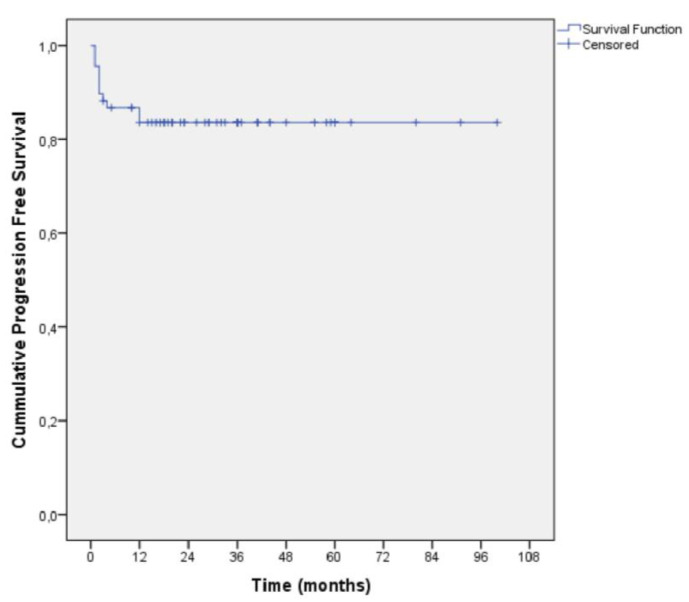
Kaplan–Meier curve for progression free survival.

Mean survival time in the total sample was 35.6 months (SD = 21.1 months) whilst the median survival time was 33 months (interquartile range: 18–49 months) (Figure 1). The recurrence free survival rate for T1a RCC was 94.7% (post secondary clinical success) and 90% (post primary clinical success). The mean progression free survival time from last ablation was 84.2 months (SE = 4.4 months). For T1a tumors, the cumulative progression free survival rate for 1, 6, 12 and 36 months were 100% (SE = 0%), 91.2% (SE = 3.7%), 91.2% (SE = 3.7%) and 87.5% (SE = 4.4%). For T1b tumors, the cumulative progression free rate for 1, 6, 12 and 36 months were 100% (SE = 0%), 63.6% (SE = 14.5%), 63.6% (SE = 14.5%) and 63.6% (SE = 14.5%).

**Figure 2:**
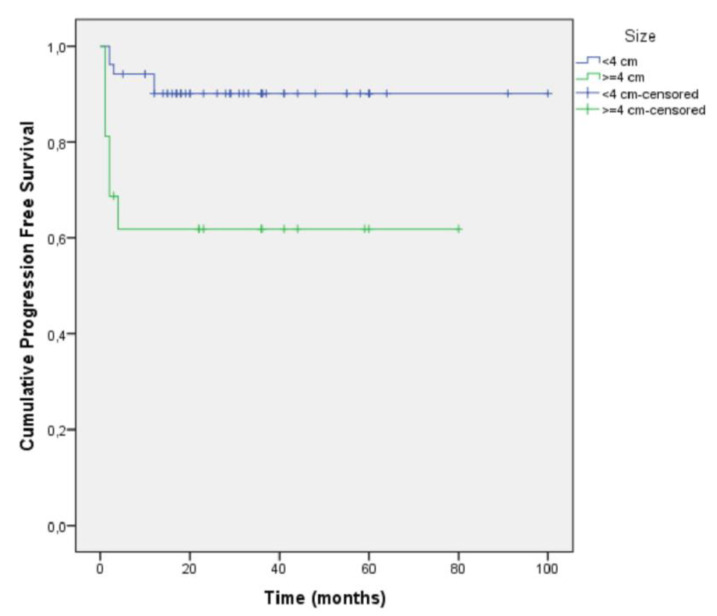
Kaplan–Meier curve for progression free survival by tumor size.

Patients of the T1b stage had a 3.72 times greater hazard for progression compared to patients of the T1a stage (Figure 2, Table 3).

Multivariate analysis showed that sex (*p* = 0.894), age (*p* = 0.376), side (*p* = 0.674), complication during ablation (*p* = 0.705) and having a second ablation (*p* = 0.670) were not associated with the progression free survival rate (Table 4).

Additionally, our univariate and multivariate analysis for T1a tumors, separated in two subgroups: those with a diameter less than 3 cm and those with a diameter greater than 3 cm, showed that there was no statistically important difference regarding tumor progression between those groups (Table 5 and Table 6).

## 4. Discussion

**Figure 3:**
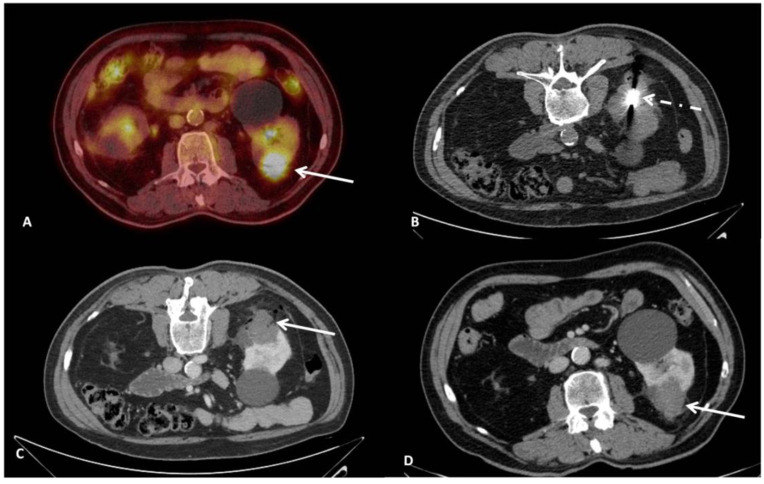
A 65 years-old male RCC patient with s solitary lesion on the left kidney. (**A**): Positron Emission Tomography-Computed Tomography (PET-CT) axial imaging illustrating the tumor (white arrow). (**B**): CT axial imaging verifying the final position of the antenna’s active tip in the lesion (white dashed arrow). (**C**): CT axial imaging post contrast medium injection in portal phase immediately post ablation illustrating the necrotic zone (white arrow). (**D**): CT axial imaging post contrast medium injection in portal phase at 12 months follow-up illustrating a lack of contrast enhancement and complete necrosis of the tumor.

Although there is an extended literature regarding mid- and long-term outcomes of RFA and CA for RCCs, MWA has been less studied and there is still a lack of data concerning long term efficacy. The present study adds to the growing number of case series showing that CT-guided percutaneous microwave ablation (Figure 3) is an efficacious and safe technique in terms of achieving local tumor control and recurrence-free response on both a per lesion and per patient basis [20,21,22,23,24,25,26,27,28,29,30,31,32,33,34,35,36].

The results of the current study showed efficient progression free (100%, 86.7%, 86.7% and 83.5% for 1, 6, 12 and 36 months, respectively), recurrence free (94.7%) and overall survival rates (mean 35.6 months), in a follow up time of 36 months. Hao et al. treated 162 patients with ultrasound guided MWA for T1a RCCs; the median follow-up time was 45.5 months and the overall occurrence of local tumor progression was 3.0% [33]. Survival rates at 1, 3 and 5 years were 98.7%, 89.5% and 82.1%, respectively [33]. Similar outcomes have been reported by Guo et al., who performed CT guided microwave ablation for T1a renal tumors in 106 patients [28]. At the first follow-up imaging study, complete response was achieved in 101 (95.3%) patients and partial response was achieved in 5 (4.7%) patients; the 1-, 2-, and 3-year local progression-free survival rates were 100.0%, 92.8% and 90.6%, respectively and 3-year overall survival were 99.0%, 97.7%, and 94.6%, respectively [28]. Several authors have demonstrated consistent outcomes to our study (Table 7).

Similar to other studies, in the present case series the treatment of RCC lesions with microwave ablation was successful and well tolerated; one major difference of the present study is that all patients were treated under local anesthesia combined with intravenous analgesia; however, this resulted in no significant differences concerning the efficacy and safety rates [21,22,23,24,25,26,27,28,29,30,34,35]. Furthermore, in tumors with a close proximity to renal pelvis/ureter, a ureteral stent was placed. Overall success rates as well as complication rates were comparable to that of other MWA (Table 6), RFA and CA studies [37,38,39,40,41,42,43]. In a recent propensity-matched analysis comparing percutaneous MWA versus laparoscopic PN for the treatment of T1a RCC, authors reported no significant differences between the two treatment arms regarding oncologic outcomes and complications [44]. In terms of minimizing invasiveness and bleeding complications, robotic partial nephrectomy has also emerged as a safe and effective surgical approach for renal tumors; until now however, there have not been prospective comparative studies against percutaneous therapies [45].

There seems to be a relation between size and local tumor control (tumor size is generally considered a survival prognostic indicator after percutaneous ablation). Several findings in the present study were noteworthy; there was no statistically significant difference regarding tumor progression/recurrence when comparing T1a tumors with a diameter <3 cm to those with a diameter between 3–4 cm (i.e., progression free survival rate was comparable among all T1a RCC patients). The results of the present study indicate that microwave ablation seems to be an efficacious technique for T1a RCC lesions, even for those at the higher (3–4 cm) size of the subgroup without any compromises upon safety. Although international guidelines advocate application of thermal ablation as an alternative option for the management of localized renal cancer <3 cm in size, the present study indicated that microwave ablation in T1a RCC patients with lesions ≥3 cm resulted in comparable overall survival and progression free survival rates to those with a tumor size <3 cm. The results of the present study can provide evidence to expand the indications of microwave ablation in treating T1a RCC in terms of tumor size.

On the other hand, LCT of MWA for T1b lesions was moderate (for 1, 6, 12 and 36 months were 100%, 63.6%, 63.6% and 63.6%, respectively), adding to the growing number of publications pointing out that either multi antennae approaches or combined therapies with trans-arterial embolization could be necessary for higher success rates [46,47,48]. Potentially, cryoablation with all its advantages including multi-probe placement, ice ball visibility and ability for ice sculpting could serve as an attractive alternative for T1b RCC lesions [49].

Current guidelines remain skeptical regarding the use of thermal ablation techniques in renal tumors with size greater than 3 cm and in T1b renal tumors, due to the increased rates of recurrence. A large Dutch series retrospectively studied the primary and secondary efficacy of percutaneous microwave ablation of histologically proven T1 renal cell carcinomas and proved that primary efficacy was significantly lower for T1b lesions (52%) compared to T1a lesions (89%). Secondary efficacy rates were not statistically different (99% and 95%, respectively) [27]. Moreover, in the study of Yu et al., the size of the tumor (>4 cm) was statistically significant for tumor progression; the technical success rate was lower in larger tumors, without this difference being statistically significant [35]. On the contrary, Shakeri et al. showed that size (>4 cm) had a significant impact on the technical success (*p* = 0.039), regardless of the location of the tumor; the size was not statistically associated with complications, progression and survival rates [26]. Hao et al. reported that although the progression free rate was lower for tumors sizing 3–4 cm in comparison to greater sizes, this difference was not proven to be statistically significant [33]. The same authors reported that recurrence rates did not affect the overall survival rates after adequate repetitive treatment [33]. The mean size in the current study was one of the greatest in the available literature. In our analysis only T1b tumors (>4 cm) were significantly associated with increased risk for progression, whereas no significant difference was observed between the subgroups of T1a tumors. Tumor size did not seem to affect the overall survival rates and it was not associated with the complication rate.

Recent trials comparing MWA, RFA and cryoablation have demonstrated significantly decreased procedure times in favor of MWA with similar complication rates and renal function changes post-procedure [50,51]. Similarly, De Cobelli et al. [43] compared percutaneous MWA and cryoablation for T1a RCC and showed comparable safety and efficacy between the two modalities. In cryoablation, the use of multiple probes and the ablation protocol (with a alternating freeze-thawing cycles) itself prolongs the procedural time; moreover, an additional advantage of the MWA is the reduced cost over cryoablation [51]. All procedures in the present study were performed under local anesthesia and were well tolerated. In current literature, there is a lack of studies comparing intraprocedural pain between different thermal techniques in renal tumors. However, studies in liver and lung tumors showed that MWA is less painful compared to RFA [52,53,54,55].

The limitations of the present study include its retrospective nature and the small number of patients with larger (T1b) tumors. Biopsy results in terms of the Fuhrman grading and histologic subtype were not taken in account. Additional limitations include a lack of comparisons to RFA, CWA or surgery.

In summary, the results of the present study show that CT guided percutaneous MWA is an effective technique for treatment of T1a renal cell carcinomas, irrespective of tumor size at this stage. T1b tumors were associated with higher progression rates, therefore a size >4 cm seems to be a significant factor affecting efficacy. Large randomized controlled studies are warranted to observe treatment effectiveness and compare the results with those of other treatment options.

## Figures and Tables

**Table 1:** Patient and tumor characteristics.

	N (%)
Gender	
Female	31 (44.9)
Male	38 (55.1)
Age (years), mean (SD)	70.4 (11.5)
Side	
Left	33 (47.0)
Right	36 (53.0)
T	
1a	57 (82.6)
1b	12 (17.4)
Size (cm), mean (SD)	3 (1.3)

**Table 2:** Analytical table with patient characteristics. M: Male, F: Female, R: Right, L: Left.

Gender	AGE (Years)	Side	Tnm	Size (cm)
F	85	L	T1aN0M0	2
Μ	21	L	T1aN0M0	1
Μ	69	L	T1bN0M0	6
Μ	65	L	T1aN0M0	4
Μ	87	R	T1aN0M0	2.7
F	66	L	T1aN0M0	2
F	77	R	T1aN0M0	3
Μ	42	L	T1aN0M0	2.2
Μ	72	R	T1aN0M0	3.1
M	69	L	T1aN0M0	1.2
F	83	R	T1bN0M0	4.6
M	79	L	T1aN0M0	2
F	82	R	T1bN0M0	4.8
F	61	R	T1aN0M0	2
Μ	45	L	T1bN0M0	4.3
F	73	R	T1aN0M0	3.6
Μ	81	R	T1aN0M0	3
Μ	77	R	T1aN0M0	4
F	62	L	T1aN0M0	2.1
F	59	R	T1bN0M0	5.9
F	81	L	T1aN0M0	3.7
F	68	R	T1aN0M0	3
F	61	L	T1aN0M0	3.5
M	71	L	T1aN0M0	2.6
M	70	R	T1aN0M0	1.7
M	80	R	T1aN0M0	1
F	66	R	T1aN0M0	2.5
Μ	79	L	T1bN0M0	4.1
F	77	R	T1aN0M0	3
M	81	R	T1aN0M0	1.8
F	71	R	T1aN0M0	2.3
F	73	L	T1aN0M0	1
M	69	L	T1aN0M0	3.7
Μ	66	R	T1aN0M0	3.5
Μ	73	R	T1bN0M0	5
F	52	L	T1aN0M0	2.5
M	91	L	T1aN0M0	1.9
Μ	84	R	T1bN0M0	5
F	61	R	T1aN0M0	2.2
M	60	L	T1aN0M0	3
M	64	L	T1aN0M0	1
Μ	76	R	T1aN0M0	2.5
F	76	R	T1aN0M0	4
F	69	R	T1aN0M0	4
F	52	L	T1aN0M0	2.4
M	65	L	T1aN0M0	2.9
M	75	L	T1aN0M0	1.6
F	82	R	T1aN0M0	3.8
F	76	R	T1aN0M0	4
M	59	L	T1aN0M0	1.4
Μ	65	L	TIbN0MO	6
F	84	L	T1aN0M0	2
F	66	R	T1aN0M0	2.1
M	77	L	T1aN0M0	2
F	68	R	T1aN0M0	3.2
M	65	L	T1aN0M0	1.5
F	70	L	T1bN0M0	5.9
F	65	R	T1aN0M0	2.4
Μ	74	R	T1bN0M0	5
Μ	62	R	T1aN0M0	3.5
F	67	R	T1aN0M0	1.8
M	72	R	T1aN0M0	3.4
F	81	L	T1bN0M0	5.6
F	83	R	T1aN0M0	3
F	78	L	T1aN0M0	1.7
M	68	L	T1aN0M0	2.8
M	72	R	T1aN0M0	2.4
Μ	70	R	T1aN0M0	2.5
M	87	L	T1aN0M0	2.7

**Table 3:** Univariate Cox regression results for disease progression.

	HR (95% CI)+	*p*	
Gender			
Females (reference)			
Males	1.01 (0.31–3.29)	0.993	
Age (years)	1.01 (0.96–1.07)	0.730	
Side			
Left (reference)			
Right	1.01 (0.29–3.48)	0.989	
T			
1a (reference)			
1b	3.72 (1.08–12.78)	0.037	
Complications			
No (reference)			
Yes	0.82(0.11–6.43)	0.853	

**Table 4:** Multivariate Cox regression results for disease progression.

	HR (95% CI)+	*p*
Gender		
Females (reference)		
Males	0.68 (0.17–2.76)	0.589
Age (years)	1.00 (0.91–1.08)	0.911
Side		
Left (reference)		
Right	0.99 (0.21–4.61)	0.990
Size (cm)		
≤4 cm		
>4 cm	7.09 (1.21–41.51)	0.030
Complications		
No (reference)		
Yes	0.37(0.43–3.24)	0.372

**Table 5:** Univariate Cox regression results for disease progression in T1a tumors.

	HR	95.0% CI		
		Lower	Upper	*p*
≤3, reference				
3.1–4	2988	0.499	17,894	0.231

**Table 6:** Multivariate Cox regression results for disease progression in T1a tumors.

	*p*	HR	95.0% CI	
			Lower	Upper
≤3				
3.1–4	0.676	1623	0.168	15,696

**Table 7:** Studies showing their results in percutaneous microwave ablation of renal cell carcinomas.

Authors	Patients	Tumor Size (Mean)	Imaging Guidance System	Follow Up	Technical Success	Clinical Effectiveness	Complications	Free Survival Rate	Overall Survival Rate	Primary Efficacy
Carafiello et al. [16]	12	2.0 cm	CT	6 m	100%	100%	0%			
Chan et al. [17]	62	25.6 mm	CT	24 m	93.50%	94%	4.8%	95%	97% at 2 years	
Klappelich et al. [18]	96	2.6 cm	CT or US	17 m	100%		9.30%	88%	91%	
Genson et al. [19]	23	2.7 cm	CT or US	12.2 m	100%	100%	4% major and 13% minor	78.80%		
Jin Yu et al. [30]	46	3.0 cm	US		98.00%		0%	100%	100%, 100%, and 97.8%	
Acosta Ruiz V. et al. [20]	93	25 mm	CT	2.1 y			5.20%			96.20%
Thompson SM et al. [21]	26	2.3 cm	CT	20.6 ± 11.6 m	100%		19.20%	96%	94%	
Shakeri S et al. [22]	56	2.5 cm	CT or US	6 m	92.80%		5.8%		96.70%	
Aarts BM et al. [23]	100	3.2 cm	CT	19 m			19%			89%
Guo et al. [24]	106	T1a	CT		100%		5.70%	100.0%, 92.8%, and 90.6% at 1-, 2-, and 3-years, respectively	99.0%, 97.7%, and 94.6% at 1-, 2-, and 3-years, respectively	
Guo et al. [25]	23	T1a	CT		87%		8.70%	100.0%, 90.9% and 90.9% at 1, 2 and 3 years, respectively	95.2%, 85.7% and 71.4% at 1, 2 and 3	
Wells et al. [31]	29	2.8 cm		12 m	96% T1a, 100% T1b		10%			
Maciolek et al. [26]	148	2.4 cm	CT or US	32 m	100%		14%	95%	96%	100%
Sui et al. [32]	31	1.92 cm	US	2 y			9.7%	96.8%		
Gao et al. [27]	41	3.6 ± 1.2 cm	CT or US	37.6 m	92.7%		100%, 89.7% and 81.5%		97.1%, 87.8% and 83.6%	
Hao et al. [29]	162	2.9 cm	us	44 m				1, 3 and 5 years were 98.7%, 89.5% and 82.1%	1, 3 and 5 years were 98.1%, 92.8% and 85.9%	
Ierardi et al. [28]	58	2.36 ± 0.9 cm	US + CT	25.7 m	100%	Major in 2 (2/58) and minor in 3 patients (3/58)	87.9% at 5 years	15.1% at 1 year	80.60%	

## Data Availability

The data presented in this study are available on request from the corresponding author. The data are not publicly available at the moment, since the present study is the basis for the PhD thesis of the first author.
